# Early Rising: Cheerful Health

**Published:** 1887-04-16

**Authors:** 


### Early Rising: Cheerful Health.

By Mrs. Hynes.

Labour, in some form or other, is undeniably necessary to man. We are told "man goeth forth to his labour until the evening" ; and it is undoubtedly right that everyone, according to his position and circumstances, should apply himself to it ; but the strength of human nature would soon be exhausted, and mind and body alike become incapable, were it not for the recuperative powers of sleep. The continual dissipation of the nervous fluid which puts the springs of brain and muscle into play, and supports the action and motion of our bodies, would soon cause its destruction were it not that these losses are continually and regularly repaired. Dr. Forbes Winslow remarks that "there is no fact more clearly established in the physiology of man than this, that the brain expends its energies and itself during the hours of wakefulness, and that these are recuperated during sleep." He also goes on to say that unless the recuperation equals the expenditure, the brain withers—this is insanity. People condemned to death by that most cruel of methods—being debarred from all sleep—died raving maniacs. We read of it in early English history, and it has been practised in China during the present age. Dr. Winslow draws three practical inferences—1st, Those who think most, who do most brain-work, require most sleep ; 2nd, That time "saved" from necessary sleep is infallibly destructive to mind, body, and estate ; 3rd, Give yourself, your children, your servants,—give all that are under you, the fullest amount of sleep they will take, by compelling them to go to bed at some regular, early hour, and to rise in the morning the moment they awake.

It is an oft-repeated anecdote of the Duke of Wellington, who was remarkable, as so many other great men have been, for his early rising, that he said, "If I find myself turning in bed I immediately turn out" ; on hearing which, his companion, a witty prelate, humorously observed "that *he* did not, for he thought 'one good turn deserved another.'"

"The early bird catches the worm," is a well-known and popular proverb, and yet it too has been cynically answered by the witty and obvious suggestion that the worm must have been a still earlier riser than the bird, or he would not have been there ready to be caught ; but in spite of all the scoffings of the indolent and self-indulgent, no reasonable being can deny that a habit of early rising, conscientiously formed and steadily persisted in, is distinctly conducive to health, happiness, usefulness, and longevity.

Doddridge says the difference between rising at five and seven o'clock in the morning, for the space of forty years, supposing a man to go to bed at the same hour at night, is nearly equivalent to the addition of ten years to a man's life. Franklin tells us that the morning hour "has gold in its mouth," and he also tritely remarks that he who rises late may trot all day, and not have overtaken his business at night. Dean Swift avers that he never knew any man come to greatness and eminence who lay in bed of a morning ; while good old Jeremy Taylor advises us to let our sleep be necessary and healthful, not idle and expensive of time beyond the needs and conveniences of nature, and he further bids us to sometimes be "curious to see the preparation which the sun makes when he is coming forth from his chambers of the east."

Again, we are told, "Rise with the lark, and with the lark to bed," and yet another oft-quoted saying is familiar in its rhyming jingle to all ears—

> "Early to bed, and early to rise,
> Makes a man healthy, wealthy, and wise."

How many thousands of times has this been repeated by wearied nurses and overtaxed mothers to the troublesome children in their charge, who beg and clamour so persistently to be allowed "to sit up—just a little longer !"

In *Paradise Lost* Milton speaks of Adam's sleep, before the Fall, as "airy light from pure digestion bred," and that he was awakened from it by the "shrill matin song of birds on every bough." He also makes our first parent say to his partner Eve, on their retiring to rest—

> "To-morrow, ere fresh morning streak the east,
> With first approach of light, we must be risen,
> And at our pleasant labour."

I have seen it argued that too late hours in bed present an index to character, and that those who indulge in this slothful habit of wasting the most beautiful and inspiring part of the day, clearly demonstrate their own tendency to self-indulgence. It betokens a feeble, inert mind, infirm of purpose, and lacking that superior vigour of will which enables the possessor to accomplish whatever his reason ordains. This weakness of character, joined as it so often is to a languid, indolent, and yet amiable and docile temperament, is the cause of so many objectless, seemingly wasted lives— lives which might have attained high honour and distinction, if only the fatal luxury of sloth had been overruled by vigour, promptitude, and decision. Solomon, the wisest of men, had much to say on this head. "Yet a little sleep,a little slumber, a little folding of the hands to sleep ; so shall thy poverty come as one that travelleth ; and thy want as an armed man." Everyone despises a sluggard, or looks upon him with a contemptuous pity, and yet there are some whom necessity compels to get up early in the morning, and to whom it is little less than a daily martyrdom. It is a duty which, *theoretically*, we all know to be good and true, but which, *practically*, not a few of us find most difficult. However, I think I have said enough to justify my strong advocacy of the really noble habit of early rising.

### SHORT AND SWEET.

A refuge in sickness, where medicine and care,
The suffering, the weary, young or old, alike share.

Paddy.

